# A Case Report: Giant Liver Hemangioma Treated with Transcatheter Embolization

**DOI:** 10.7759/cureus.65927

**Published:** 2024-08-01

**Authors:** Sudhanshu Tonpe, Himandri Warbhe, Pankaj Banode, Sneha Bandi, Manasa Suryadevara, Swaroop Reddy Guggella

**Affiliations:** 1 Department of Interventional Radiology, Jawaharlal Nehru Medical College, Datta Meghe Institute of Higher Education and Research, Wardha, IND; 2 Department of Respiratory Medicine, Jawaharlal Nehru Medical College, Datta Meghe Institute of Higher Education and Research, Wardha, IND; 3 Department of Radiology, Siddhartha Medical College, Vijayawada, IND; 4 Department of Radiodiagnosis, Jawaharlal Nehru Medical College, Datta Meghe Institute of Higher Education and Research, Wardha, IND; 5 Department of Radiodiagnosis, Datta Meghe Institute of Higher Education and Reasearch, Wardha, IND; 6 Department of Radiology, Sainath Hospital and Prime Diagnostics, Ananthapur, IND

**Keywords:** endovascular embolization, bleomycin, lipidol, angiography, giant hepatic hemangioma

## Abstract

When treating patients with a massive cavernous hemangioma of the liver that requires nonsurgical therapy, transcatheter arterial embolization has proven to be an effective technique. Significant advantages include the ability to obliterate the vascular supply of these lesions and the minimally invasive nature compared to surgery. A 65-year-old woman arrived at our hospital complaining primarily of stomach pain that had been there for six months. The patient had a hard lump in the right hypochondrium on clinical examination. Ultrasound showed a large, well-defined, heterogeneous lesion with central necrotic areas, with the rest of the liver parenchyma having normal echotexture and flow in the portal vein. The 65-year-old woman's primary complaint upon arrival at our hospital was a stomach ache that had been there for six months. The results of the liver function test were normal. Upon presumptive identification of a significant hepatic hemangioma, the patient was brought to the angio-suite for angiography and proper hepatic artery embolization. Considering the patient's age, the severity of the lesion, and its highly vascular character, endovascular embolization of the proper hepatic artery using lipidol and bleomycin was performed. The patient was discharged after two days in the hospital, administered antibiotics, and advised to follow up after 15 days. Liver function after embolization was within normal limits. The patient had no symptoms after a follow-up at three months. Therefore, endovascular embolization with lipidol and bleomycin is a safe and effective method to obliterate the vascular supply to the lesion, prevent catastrophic bleeding, and provide symptomatic relief to the patient.

## Introduction

When treating patients with a massive cavernous hemangioma of the liver that requires nonsurgical intervention, transcatheter artery embolization has proven to be a successful technique. Significant advantages include the ability to obliterate the vascular supply of these lesions and the minimally invasive nature compared to surgery [1,2]. This is the case report of a patient treated with endovascular transcatheter embolization.

## Case presentation

Patient information and clinical findings

**Figure 1:**
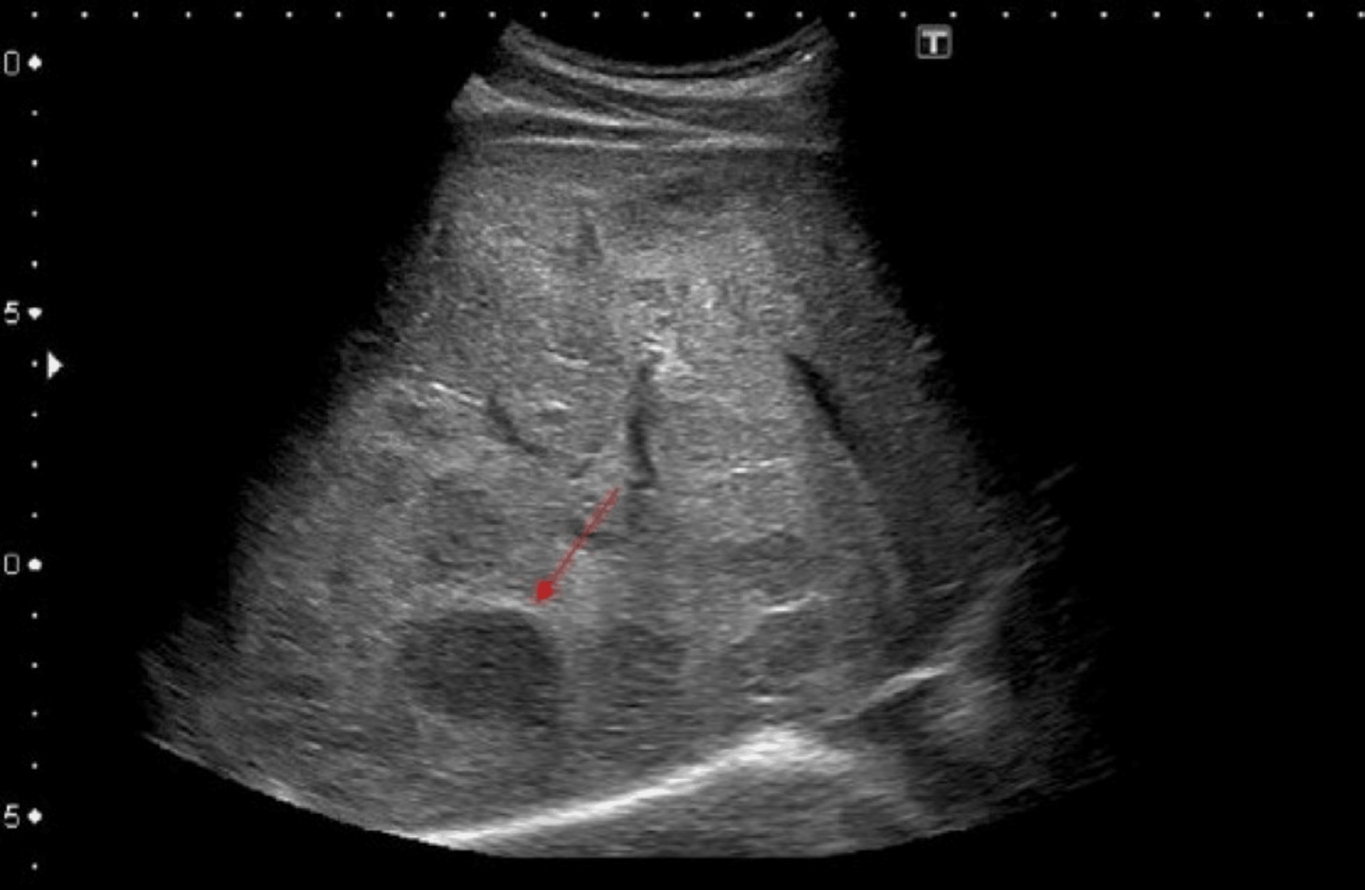
Ultrasound of the liver showing a large, well-defined, heterogenous lesion with central necrotic areas (red arrow), with the rest of the liver parenchyma of normal echotexture

A 65-year-old female came to our hospital with a chief complaint of dull abdominal pain for six months localized in the right upper quadrant. The patient had a hard lump in the right hypochondrium on clinical examination. A significant, well-defined, heterogeneous lesion with central necrotic regions was seen on ultrasound, although the liver parenchyma's echotexture and portal vein flow were normal throughout (Figure 1).

**Figure 2:**
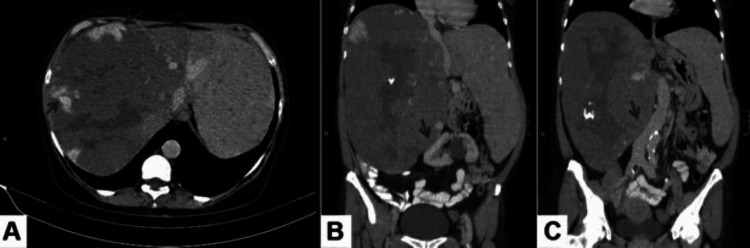
CECT scan of the abdomen in axial (A) and coronal (B, C) sections. The lesion measures 25x15 cm, which shows nodular enhancement in the periphery corresponding to the blood pool (grey arrow) CECT: contrast-enhanced computed tomography

The lesion spanned 25x15 cm on a contrast-enhanced computed tomography (CECT) scan of the abdomen and a nodular enhancement was seen in the periphery corresponding to the blood pool (Figure 2).

The results of the liver function test were normal. The patient's electrocardiogram (ECG), pulmonary function, and echocardiogram were normal. The patient had a history of hypertension but no history of diabetes mellitus.

**Figure 3:**
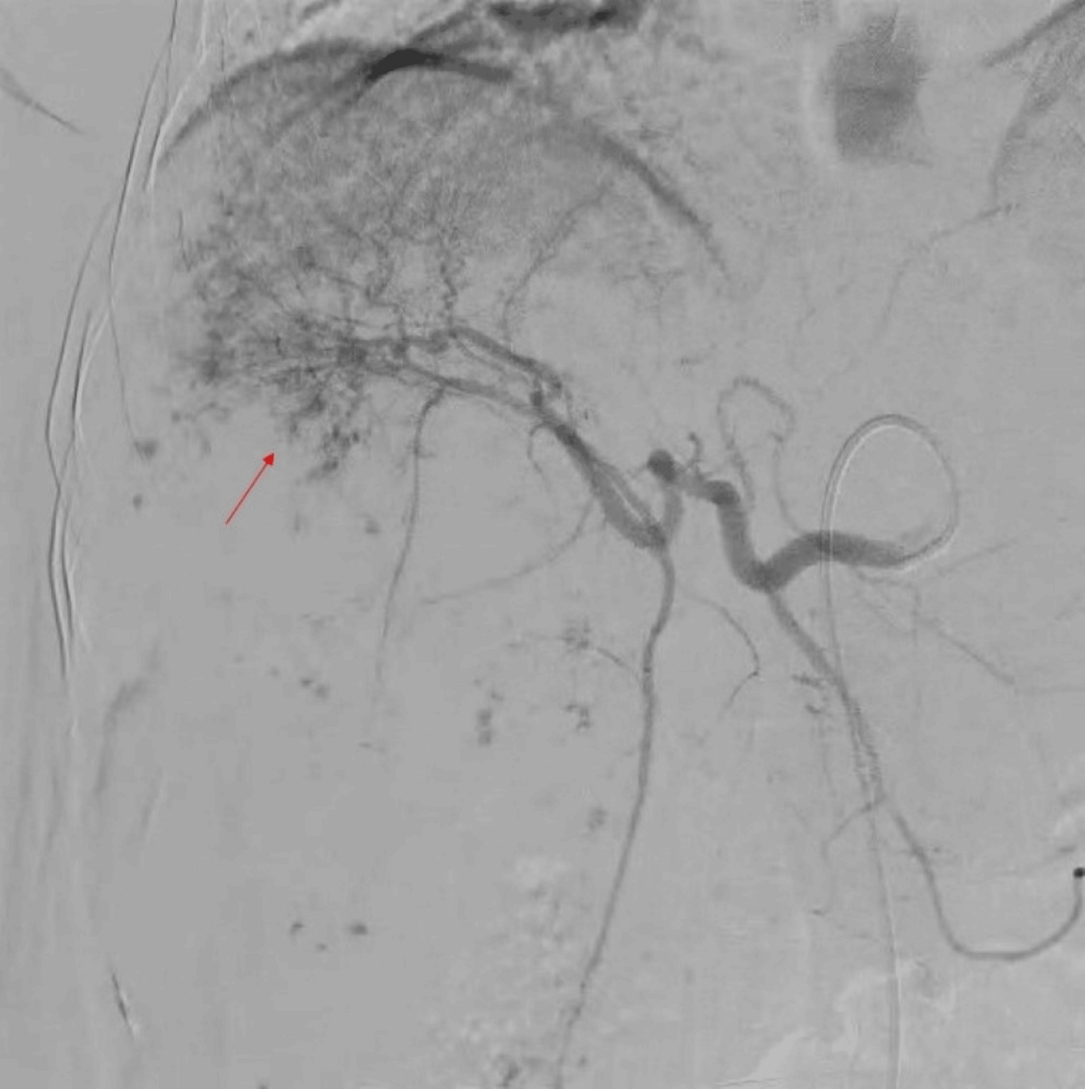
Angiography after selective cannulation of the common hepatic artery demonstrating a large vascular lesion completely occupying the right lobe of the liver with nodular vascular channels (red arrow)

After a preliminary diagnosis of a massive hepatic hemangioma, the patient underwent an angiography, and the appropriate hepatic artery was embolized (Figure 3).

Therapeutic intervention

Surgical Procedure

**Figure 4:**
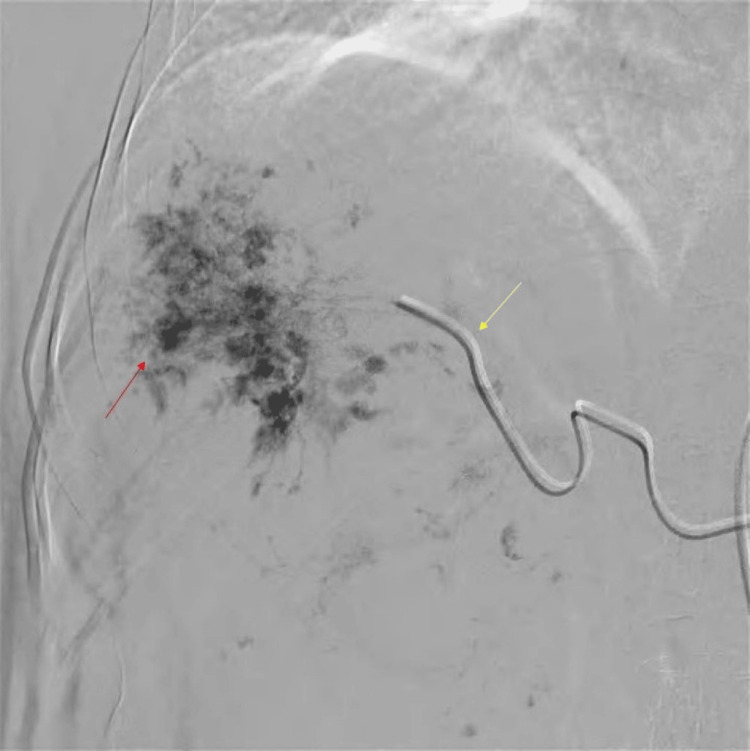
Angiography after super-selective cannulation (yellow arrow) of the right hepatic artery demonstrating a large vascular lesion completely occupying the right lobe of the liver with nodular vascular channels (red arrow)

A 5-French (Fr) Cobra catheter (Cook Medical, Bloomington, USA) was selectively cannulated into the common hepatic artery under fluoroscopic control with the aid of a 5-Fr short femoral sheath inserted into the right femoral artery, and the patient was placed supine on the operating table in the angiosuite. The patient remained conscious during the entire procedure. A 2.4-Fr microcatheter (Progreat, Terumo Corporation, Tokyo, Japan) was coaxially positioned in the proper hepatic artery through the Cobra catheter, and an angiogram was obtained (Figure 4).

**Figure 5:**
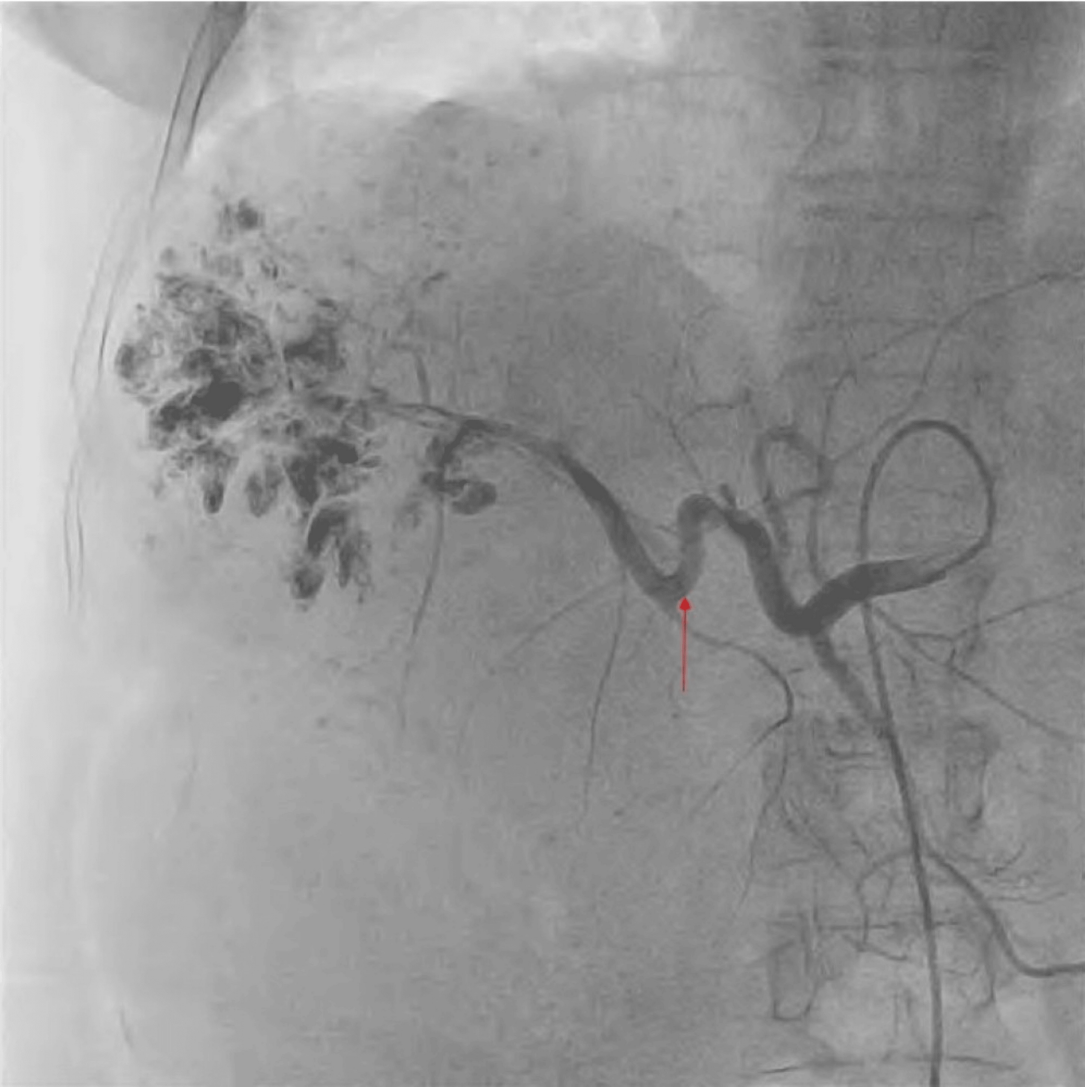
Angiogram obtained shows complete obliteration of flow to the right hepatic artery with reflux of contrast in the common hepatic artery (red arrow)

Around 4 mL of aqueous bleomycin was mixed with 10 mL of lipiodol and administered through the microcatheter. Gelfoam was topped up at the end, and an angiogram was obtained (Figure 5).

Digital subtraction angiography (DSA) showed complete obliteration of flow to the proper hepatic artery with reflux of contrast in the common hepatic artery. After removing the microcatheter, the right femoral artery was directly compressed to cause hemostasis.

Follow-up and outcomes

The patient was released from the intensive care unit on the first postoperative day following the procedure, and on the second day, they were released from the hospital. The patient had no complaints in the second week and three months after the procedure.

## Discussion

Cavernous liver hemangiomas are characterized as giant when exceeding 5 cm in diameter [3]. It is generally accepted that liver hemangiomas need no treatment since they are benign lesions showing no growth over time [2,4]. However, giant hemangiomas are often associated with clinical symptoms and the development of complications, among which rupture with intraabdominal hemorrhage is a rare but life-threatening condition developing after the trauma but also spontaneously [1, 5-9]. Therefore, in symptomatic cases, treatment is considered with endovascular management. Surgical resection is the radical treatment with a reasonably low mortality risk, of less than 4.3% mortality, in elective operations in experienced centers [2,3,10]. Patients with comorbidities and coexisting diseases may be high-risk surgical candidates who may need liver transplants. Transcatheter arterial embolization is a valuable technique for managing giant cavernous hemangioma of the liver in nonsurgical patients. Significant advantages include the ability to obliterate the vascular supply of these lesions and the minimally invasive nature compared to surgery.

In our instance, the bleomycin-lipiodol emulsion was employed as an embolizing agent to treat gigantic cavernous hepatic hemangiomas. This agent damages the endothelium of the sinusoids of the hemangiomas by reducing the DNA production of cancer cells and cutting off the DNA chain [11].

Alternative embolic agents that can be used

Embospheres have the advantage of long-term occlusive properties, biocompatibility, and the ability to reach even the tiny sinusoidal spaces of the hemangiomas; however, it is expensive. Liquid embolic agents like ethanol or acrylic glue have the advantage of causing blood plasma protein coagulation immediately upon infusion; however, reflux embolization might result in severe consequences. Gelfoam can be used as a temporary embolic material.

The most important complication related to embolization is necrosis or infarction. Selective embolization and portal vein patency are essential parameters that preclude the development of these complications. In addition, we used antibiotic prophylaxis in all our patients. Distant embolization may develop when arteriovenous shunts are present (in one of our patients precluded from embolization), but they are infrequently encountered in liver hemangiomas [12]. The mesoderm gives rise to cavernous liver hemangiomas, which comprise several tiny, interconnected vascular compartments with different diameters [13,14]. Without any muscle or elastic tissue layers, the sinus wall is composed of a single layer of immature endothelial cells.

Fibrous septae of variable thickness are also present [13,14]. The hepatic artery is typically the arterial supply of cavernous liver hemangiomas. On improved CECT and angiography, arteriovenous shunts have only been seen in a few instances [12]. The function of the embolic material entering the sinuses is to slow blood flow further and propagate local thrombosis. Nevertheless, none of the abovementioned substances directly affect the sinus endothelium, and because the liver has two blood supplies, revascularization may be brought about via the portal flow system. These last two factors may explain the absence of a significant reduction in the size described in most series post-embolization [15]. A significant decrease is represented by Graham et al. [16].

## Conclusions

Transcatheter arterial embolization is a successful and minimally invasive technique for treating patients with massive cavernous hemangiomas of the liver. It effectively reduces tumor size and alleviates symptoms while offering a favorable safety profile compared to surgical options. Careful patient selection, preparation, and follow-up are crucial for optimizing outcomes.
